# Locality based Approach for containment of COVID-19 Infections in Pakistan’s High risk Districts-ICT

**DOI:** 10.1093/eurpub/ckac131.190

**Published:** 2022-10-25

**Authors:** N Noreen, I Naveed, F Bashir

**Affiliations:** 1 Directorate of Central Health Establishment, Ministry of National Health Regulations, Islamabad, Pakistan; 2 PIMS, Covid Ward, Islamabad, Pakistan; 3 PHRC, NIH, Islamabad, Pakistan

## Abstract

**Background:**

In May 2020, considering gradual restoration of all economical activities the Government of Pakistan updated containment strategy from locking down the whole country to locking down high-risk areas to mitigate COVID-19 spread. All districts having ≥300 cases/100,000 population. COVID-19 case incidence and test positivity rates by real-time RT-PCR before and after zonal lockdown were compared to assess whether the locality-based lockdowns can be used as an alternative to country lockdown to contain COVID-19 spread.

**Methods:**

Smart lockdowns were implemented in ten localities in the Islamabad Capital Territory (ICT), having a population of 60,000 from 12 May 2020 to June 3, 2020. Movements were restricted. Entry and exit points were guarded by police. Any person with symptoms of fever, cough, or sore throat tested by real-time RT-PCR methods and reported within 24 hours of collection. To compare the rate of active cases and positivity rate by weeks, we performed a z-test for two proportions and set p < 0.05 as the level of significance.

**Results:**

The red zone had 60,000 persons in 2.00 square kilometers. The rate of active COVID-19 cases significantly decreased (p < 0.0001) during intervention from 300/100,000 population pre-containment time to 22/100,000 population after the first three weeks of lockdown. The COVID-19 positivity rate also decreased significantly (p < 0.0001) from 24% (24/78) pre-containment to 5.3% during containment. A total of 3800 people were tested in the following three weeks of intervention and 26 cases were detected.

**Conclusions:**

The smart lockdowns approach reduced COVID-19 transmission in the ICT district. This type of intervention was recommended to reduce the COVID-19 infection spread

**Key messages:**

• Reduced COVID-19 transmission in the ICT district.

• Keeping balance between life and economy.

